# Gestational weight gain in women with pre-pregnancy overweight or obesity and anthropometry of infants at birth

**DOI:** 10.3389/fped.2023.1142920

**Published:** 2023-03-16

**Authors:** Christina Sonne Mogensen, Helle Zingenberg, Jens Svare, Arne Astrup, Faidon Magkos, Nina Rica Wium Geiker

**Affiliations:** ^1^ Department of Nutrition, Exercise and Sports, University of Copenhagen, Frederiksberg, Denmark; ^2^ Department of Obstetrics and Gynecology, Copenhagen University Hospital Herlev-Gentofte, Herlev, Denmark; ^3^ Dietetic and Clinical Nutrition Research Unit, Copenhagen University Hospital Herlev-Gentofte, Herlev, Denmark

**Keywords:** newborn, body weight (BW), BMI z-score (zBMI), pre-conception, pregnancy outcomes

## Abstract

**Objective:**

To examine the association of gestational weight gain (GWG) among women with pre-pregnancy overweight or obesity with infant weight and BMI z-score at birth.

**Methods:**

This study is a secondary analysis of a randomized controlled trial including data from 208 infants at birth born by mothers with pre-pregnancy BMI between 28 and 45 kg/m^2^ who completed the APPROACH study (randomized to a high-protein low-glycemic index diet or a moderate-protein moderate-glycemic index diet). This analysis pooled the two diet treatment groups together and data were analyzed using a linear mixed model.

**Results:**

Limiting GWG by 1 kg was associated with lower birthweight (−16 g, *P* = 0.003), BMI z-score (−0.03SD, *P* = 0.019), weight z-score (−0.03SD, *P* = 0.004), and infant abdominal circumference (−0.06 cm, *P* = 0.039). Infants born by mothers whose GWG was ≤9 kg weighed less (122 g, 95% CI: 6–249, *P* = 0.040), had similar BMI z-score (0.2SD, 95% CI: −0.06 to 0.55, *P* = 0.120), and lower incidence of emergency cesarean deliveries (11.5% vs. 23.1%, *P* = 0.044) compared to infants born by mothers whose GWG was >9 kg. When women were classified into GWG quartiles, women in Q1 (GWG range: −7.0 to 3.2 kg) gave birth to smaller infants (3,420 g, *P* = 0.015) with lower BMI z-score (−0.5SD, *P* = 0.041) than women in Q2 (3.3–7.1 kg), Q3 (7.2–10.9 kg) and Q4 (11.1–30.2 kg).

**Conclusions:**

Limiting GWG among women with pre-pregnancy overweight or obesity was associated with lower infant weight, BMI z-score, weight z-score, and abdominal circumference at birth. Moreover, GWG below the Institute of Medicine guideline of a maximum of 9 kg was associated with lower birthweight and fewer emergency cesarean deliveries.

## Introduction

Maternal obesity before pregnancy is positively associated with increased risk of developing serious complications related to pregnancy and childbirth (1, 2) as well as an increased risk of infant and child obesity (3). In addition to pre-pregnancy weight, excessive gestational weight gain (GWG) is also directly associated with increased risk of high birthweight (4) and subsequent obesity during childhood and adulthood (5–7). Having overweight or obesity during the early years of life tracks into having obesity later in life (8, 9) and increases the risk for cardiovascular disease (10) and diabetes (11) in adulthood. To prevent complications related to maternal and infant outcomes, the Institute of Medicine (IOM) recommends women with normal body weight before pregnancy to gain between 11.5–16 kg during pregnancy, women with overweight to gain 7–11.5 kg whereas women with obesity to gain 5–9 kg (12). Nevertheless, despite this recommendation, about 58% of women in the Danish National Birth Cohort with pre-pregnancy obesity gained 10 kg or more (13).

Lifestyle interventions during pregnancy that focus on limiting GWG generally produce only small reductions in GWG and have no significant effects on infant and maternal health (14–17). Therefore, the objective of this analysis was to evaluate the relationship between GWG among women with pre-pregnancy overweight or obesity (dichotomized according to IOM recommendations; categorized in quartiles; or treated as a continuous variable) and infant weight and BMI z-score at birth. We hypothesized that limiting GWG, particularly below 9 kg, would be associated with lower infant birthweight and BMI z-score compared with GWG above 9 kg.

## Materials and methods

### Study design

This study is a secondary analysis of the APPROACH (an optimized programming of healthy children) study, which was a randomized controlled trial conducted from January 2014 to December 2017 at a public hospital in the Greater Copenhagen Area (Copenhagen University Hospital Herlev-Gentofte, Denmark). The APPROACH study aimed to assess the effects of a high protein low glycemic index (HPLGI) diet compared to a moderate-protein moderate-glycemic index (MPMGI) diet on GWG, birthweight, and risk of gestational complications in pregnant women with obesity. No significant differences between the HPLGI diet and the MPMGI diet were found in birthweight and other anthropometric outcomes of infants at birth (18); therefore, infants born by mothers following the two experimental diets were pooled for this analysis. All study procedures were conducted in accordance with the Helsinki II Declaration. Women participating in the study received both written and oral information about the study before signing an informed consent. The APPROACH study was approved by the Ethical Committee of the Capital Region of Denmark (H-3-2013-119) and was registered at ClinicalTrials.gov (NCT01894139). A more detailed description of the APPROACH study design and methodology can be found elsewhere (18).

### Participants

In the APPROACH study, singleton pregnant women (from 11 + 4 to 13 + 6 weeks of gestation) were recruited at their trans-nuchal scan. They were eligible to participate in the APPROACH study if they were older than 18 years of age and had a pre-pregnancy BMI between 28 and 45 kg/m^2^. Women were excluded if they: had multiple pregnancies, were allergic or intolerant to dairy products or fish, had a weight loss of >10 kg during the past year, had excessive alcohol consumption (>14 units of alcohol per week), or drug abuse, had underlying disorders that were evaluated to interfere with the intervention or had gestational diabetes mellitus.

### Intervention

The women were randomized in a 1:1 ratio to one of two experimental diets: (i) a diet high in protein content and low in glycemic index (HPLGI), or (ii) a diet that followed the Nordic Nutritional Recommendations with no instructions on the glycemic index (MPMGI). During pregnancy, women randomized to the HPLGI diet consumed 25% of their energy from protein with a low glycemic index (∼45 units) whereas those randomized to the MPMGI diet consumed 18% of their energy from protein with a moderate glycemic index (∼54 units).

Both the HPLGI diet and the MPMGI diet were consumed *ad libitum* and the recommended food servings were based on the individual calorie requirements for limiting GWG. Calorie requirements were estimated as basal metabolic rate, calculated by the Harris-Benedict equation (19), multiplied by a physical activity level of 1.3. During the study, participants received seven group-based dietary sessions and two individual dietary consultations. Dietary intake was assessed by a 24-hour recall during gestational weeks 21 and 32 and by a food frequency questionnaire during gestational weeks 15, 28, and 36.

### Clinical measurements

#### Maternal measurements

Gestational weight gain was calculated as the last measured body weight before birth (up to 7 days before birth) using a medical scale (Tanita, Illinois, USA) minus pre-pregnancy weight. Pre-pregnancy weight was self-reported or obtained from the first weighing by the women's general practitioner. Height was measured at the screening visit to the nearest 0.5 cm on a wall-mounted stadiometer (Seca, Germany). Parity information was obtained by questionnaires, and data on pregnancy complications were obtained from the medical registry.

#### Infant anthropometric measurements

Anthropometric measurements of the infants were obtained by midwives at birth. The length was measured to the nearest 0.5 cm by using a non-elastic measuring tape, body weight was measured to the nearest 10 grams by a medical beam scale (Tanita, Illinois, USA), and head-, abdominal-, upper arm- and thigh circumferences were measured to the nearest 0.1 cm by using a non-elastic measuring tape. Z-scores were calculated according to World Health Organisation 2009 standards (20) for sex-specific BMI-for-age, weight-for-age, and length-for-age. Gestational age was calculated as the number of days estimated from ultrasound fetal biometrics at their nuchal translucency scan (in gestational weeks+ days: 11 + 4 to 13 + 6) to birth determined by the date of delivery.

#### Complications

Pre-eclampsia was defined as systolic blood pressure ≥140 mmHg or diastolic blood pressure ≥90 mmHg in combination with proteinuria. Pregnancy-induced hypertension was defined as systolic blood pressure ≥140 mmHg or diastolic blood pressure ≥90 mmHg. The mode of delivery was defined as vaginal delivery, planned cesarean delivery, or emergency cesarean delivery.

Predefined neonatal outcomes included (i) gestational age at delivery earlier than 37 weeks or later than 41 weeks of gestation, (ii) low birthweight ≤2.5 kg or high birthweight ≥4.0 kg.

The incidence of pregnancy complications (pre-eclampsia, pregnancy-induced hypertension, and cesarean delivery) and the incidence of neonatal outcomes (preterm delivery, prolonged pregnancy delivery, small-for-gestational-age, and large-for-gestational-age) was described as pregnancy complications in this study. Small-for-gestational-age was defined as birthweight less than the standard 10th percentile of birthweight for sex and gestational age and large-for-gestational-age was defined as birthweight above the standard 90th percentile of birthweight for sex and gestational age (21).

### Statistical analysis

The primary outcomes were infant weight and BMI z-score at birth. In the Danish healthcare system, midwives measure infant body weight, length, and abdominal circumference at birth. Head circumference, arm circumference, and thigh circumference were additional measurements for the APPROACH study, which resulted in incomplete data for these parameters due to the priority of tasks and time availability of midwives at the hospital.

We reported previously that the HPLGI diet did not significantly affect infant birthweight and other anthropometric outcomes at birth relative to the MPMGI diet (18). Therefore, this analysis pooled the two diet treatment groups together and focused on the relationship of GWG with infant birthweight and anthropometric outcomes at birth.

Available case analyses were carried out. Data were tested for normality, and analyzed by using linear mixed models using the statistical program R, version 3.6.1 (22). Descriptive characteristics are presented as means (SD) or as adjusted means (SE). We conducted three types of analyses: (i) grouping women into those whose GWG was above or below the IOM recommended maximum of 9 kg, (ii) grouping women into GWG quartiles based on observed data to identify a potential threshold other than the recommended, and (iii) treating GWG as a continuous variable. Pregnancy complications were evaluated using a chi-square test. All analyses were adjusted for maternal pre-pregnancy BMI, parity, infant sex (except for the z-scores which are sex-specific), and gestational age. To compare differences between the GWG quartiles, analysis of covariance was used (ANCOVA) with Tukey's post-hoc tests.

Mean differences between groups and their 95% confidence intervals (95% CI) were computed and a significance level of 0.05 was used.

## Results

A total of 830 eligible pregnant women signed the informed consent and received both written and oral information about the study. A total of 279 women were enrolled and 209 (75%) completed the study and gave birth to 208 infants (one stillborn).

Women who completed the study and gave birth to an infant had a pre-pregnancy BMI [mean (SD)] of 34.0 (3.9) kg/m^2^. Gestational weight gain ranged from −7.0 to 30.2 kg, with a mean of 7.3 kg and a median of 7.1 kg. A total of 20 women had weight loss during pregnancy with a mean of −2.3 (1.7) kg.

### Gestational weight gain above or below 9 kg

Of 208 women, 78 women (38%) gained >9 kg, on average (SD) 13.2 (3.7) kg, while 130 women (62%) gained ≤9 kg, on average 3.8 (3.6) kg (Table 1).

**Table 1:** Infant outcomes and incidence of complications according to gestational weight gain (GWG) below or above the recommended maximum amount of 9 kg.

	GWG ≤ 9 kg (*N* = 130)		GWG > 9 kg (*N* = 78)		Difference (GWG > 9 kg - GWG ≤ 9 kg)	*P*-value
Maternal GWG, mean (SD), kg	130	3.8 (3.6)	78	13.2 (3.7)	9.4 (8.3;10.4)	<0.001
GWG range, min;max, kg	130	−7.0;9.0	78	9.1;30.2		
Pre-pregnancy BMI, mean (SD)	130	34.5 (4.1)	78	33.1 (3.3)	−1.49 (−2.6;0.41)	0.007
Parity, %	130	0.6 (0.7)	77	0.5 (0.7)		0.506
0	74	57	41	53		
1	21	32	30	39		
≥2	15	11	6	8		
Infant outcomes						
Gestational age, mean (SE), days	130	278 (1.3)	78	281 (1.7)	3.0 (−2.2;7.2)	0.165
Sex, no. (%), girls	130	59 (45)	78	35 (45)		
Length, mean (SE), cm	129	51.6 (0.2)	78	51.9 (0.2)	0.2 (−0.3;0.8)	0.402
Weight, mean (SE), g	129	3,503 (37)	78	3,631 (49)	122 (6;249)	0.040
Length z-score, mean (SE), SD	129	1.12 (0.1)	78	1.25 (0.1)	0.13 (−0.17;0.42)	0.400
Weight z-score, mean (SE), SD	129	0.39 (0.1)	78	0.63 (0.1)	0.24 (−0.01;0.50)	0.059
BMI z-score, mean (SE), SD	129	−0.31 (0.1)	78	0.06 (0.1)	0.2 (−0.06;0.55)	0.120
Head circumference (cm), mean (SE), cm	124	34.7 (0.1)	77	34.9 (0.2)	0.2 (−-0.2;0.6)	0.294
Abdominal circumference, mean (SE), cm	118	33.2 (0.2)	74	33.7 (0.2)	0.5 (−0.1;1.2)	0.089
Arm circumference, mean (SE), cm	77	11.5 (0.1)	45	12.2 (0.2)	0.7 (0.2;1.2)	0.004
Thigh circumference, mean (SE), cm	79	16.6 (0.2)	45	16.8 (0.3)	0.3 (−0.4;0.9)	0.398
Incidence of pregnancy complications						
Pre-eclampsia, no. (%)	123	1 (0.8)	75	1 (1.3)		1.000
Pregnancy-induced hypertension, no. (%)	130	1 (0.8)	78	0 (0.0)		1.000
Pre-term delivery <37 weeks, no. (%)	122	7 (5.4)	75	3 (3.8)		0.858
Prolonged pregnancy >41 weeks, no. (%)	130	39 (30.0)	78	23 (29.4)		1.000
Emergency cesarean delivery, no. (%)	130	15 (11.5)	78	18 (23.1)		0.044
Planned cesarean delivery, no. (%)	130	12 (9.2)	78	1 (1.3)		0.046
Total cesarean delivery, no. (%)	130	27 (20.8)	78	19 (24.4)		0.666
Large-for-gestational-age (90th percentile), no (%)	129	25 (19.4)	78	22 (28.2)		0.194
Small-for-gestational-age (10th percentile), no (%)	129	7 (5.4)	78	1 (1.3)		0.230
High birthweight >4,000 g, no (%)	129	21 (16.3)	78	17 (21.8)		0.419
Low birthweight <2,500 g, no (%)	129	6 (4.7)	78	3 (3.9)		1.000

Data are presented as indicated in the first column, except for differences between groups, which are presented as means with 95% confidence intervals. The analysis is adjusted for maternal pre-pregnancy BMI, parity, and infant gestational age and sex (except for z-scores, which are not adjusted for sex as z-scores are already stratified by sex).

Infants born by women with GWG ≤9 kg weighed 122 g (95% CI: 6–249; *P* = 0.040) less at birth, but there were no significant differences in BMI or length z-scores (Table 1).

A total of 176 pregnancy complications were registered, which included neonatal outcomes. Women with GWG ≤9 kg had a lower incidence of emergency cesarean delivery compared with women gaining >9 kg (11.5% vs. 23.1% respectively; *P* = 0.044), but there was a higher number of planned cesarean deliveries (9.2% vs. 1.3%, respectively; *P* = 0.046). There were no significant differences in the incidence of total cesarean delivery between groups or the frequency of other pregnancy complications (Table 1).

### Gestational weight gain quartiles

Characteristics of the infants across maternal GWG quartiles are presented in Table 2. Infants born by mothers with GWG <3.3 kg (Q1) had a significantly lower birthweight, weight z-score, and BMI z-score compared with infants born by mothers who gained >3.3 kg and up to 30.2 kg during pregnancy (Q2, Q3, and Q4). Nevertheless, there were no significant differences between Q2, Q3, and Q4. Moreover, infants born by women in Q1 had significantly lower abdominal circumferences compared with those born by women in Q4. 11.5% of infants in Q1 were born small-for-gestational age which was significantly more compared to Q2 and Q4 (1.89%, *P* = 0.047 and 0%, *P* 0.012) (Table 2).

**Table 2:** Infant outcomes and incidence of complications according to gestational weight gain (GWG) quartiles.

	Q1	Q2	Q3	Q4	*P*-value
*N*	52	53	51	52	
Maternal GWG, mean (SD), kg	0.2 (2.4)	5.2 (1.3)	9.1 (1.0)	14.8 (3.5)	<0.001
GWG range, min;max, kg	−7.0;3.2	3.3;7.1	7.2;10.9	11.1;30.2	
Infant outcomes					
Gestage, mean (SE). days	275 (2.1)^c^	280 (2.1)^a^	280 (2.1)^b^	283 (2.1)^c^	0.013
Sex, no. (%), girls	26 (50)	19 (36)	25 (49)	24 (46)	0.827
Length, mean (SE), cm	51.5 (0.3)	51.7 (0.3)	51.8 (0.3)	51.9 (0.3)	0.290
Weight, mean (SE), g	3,420 (59.7)^a,b,c^	3,564 (58.2)^a^	3,586 (59.8)^b^	3,636 (59.3)^c^	0.015
Length z-score, mean (SE), SD	1.1 (0.1)	1.2 (0.1)	1.2 (0.1)	1.3 (0.1)	0.288
Weight z-score, mean (SE), SD	0.2 (0.1)^a,b,c^	0.5 (0.1)^a^	0.6 (0.1)^b^	0.6 (0.1)^c^	0.018
BMI z-score, mean (SE), SD	−0.5 (0.2)^a,b,c^	−0.2 (0.1)^a^	−0.1 (0.2)^b^	0.0 (0.1)^c^	0.041
Head circumference mean (SE), cm	34.5 (0.2)	34.9 (0.2)	34.8 (0.2)	35.0 (0.2)	0.156
Abdominal circumference mean (SE), cm	32.7 (0.3)^c^	33.6 (0.3)^a^	33.4 (0.3)^b^	33.7 (0.3)^c^	0.040
Arm circumference mean (SE), cm	11.4 (0.2)^c^	11.5 (0.2)^a^	12.1 (0.2)^b^	12.2 (0.2)^c^	0.005
Thigh circumference mean (SE), cm	16.5 (0.3)	16.4 (0.3)	17.0 (0.3)	16.7 (0.3)	0.401
Incidence of pregnancy complications					
Pre-eclampsia, no. (%)	0 (0.0)	1 (2.0)	1 (2.0)	0 (0.0)	0.576
Pregnancy-induced hypertension, no. (%)	1 (2.0)	0 (0.0)	0 (0.0)	0 (0.0)	0.389
Pre-term delivery <37 weeks, no. (%)	4 (7.7)	3 (5.7)	2 (4.0)	1 (2.0)	0.565
Prolonged pregnancy >41 weeks, no. (%)	11 (21.2)	21 (39.6)	12 (23.5)	18 (34.6)	
Emergency cesarean delivery, no. (%)	5 (9.6)	8 (15.1)	8 (15.7)	12 (23.1)	0.312
Planned cesarean delivery, no. (%)	4 (7.7)	5 (9.4)	3 (5.9)	1 (1.9)	0.428
Total cesarean delivery, no. (%)	9 (17.3)	13 (24.5)	11 (21.6)	13 (25.0)	0.768
Large-for-gestational-age (90th percentile), no (%)	7 (13.5)	13 (24.5)	10 (20.0)	17 (32.7)	0.122
Small-for-gestational-age (10th percentile), no (%)	6 (11.5) ^a,c^	1 (1.9) ^a^	1 (2.0) ^b^	0 (0.0) ^c^	0.010
High birthweight >4,000 g, no (%)	6 (11.5)	12 (22.6)	7 (14.0)	13 (25.0)	0.219
Low birthweight <2,500 g, no (%)	4 (7.7)	2 (3.8)	2 (4.0)	1 (1.9)	0.534

Data are presented as indicated in the first column, except for differences between groups, which are presented as means with 95% confidence intervals. The analysis is adjusted for maternal pre-pregnancy BMI, parity, and infant gestational age and sex (except for z-scores, which are not adjusted for sex as z-scores are already stratified by sex).

Quartiles that have the same superscript are significantly different from each other.

### Associations between gestational weight gain and birth outcomes

**Figure 1:**
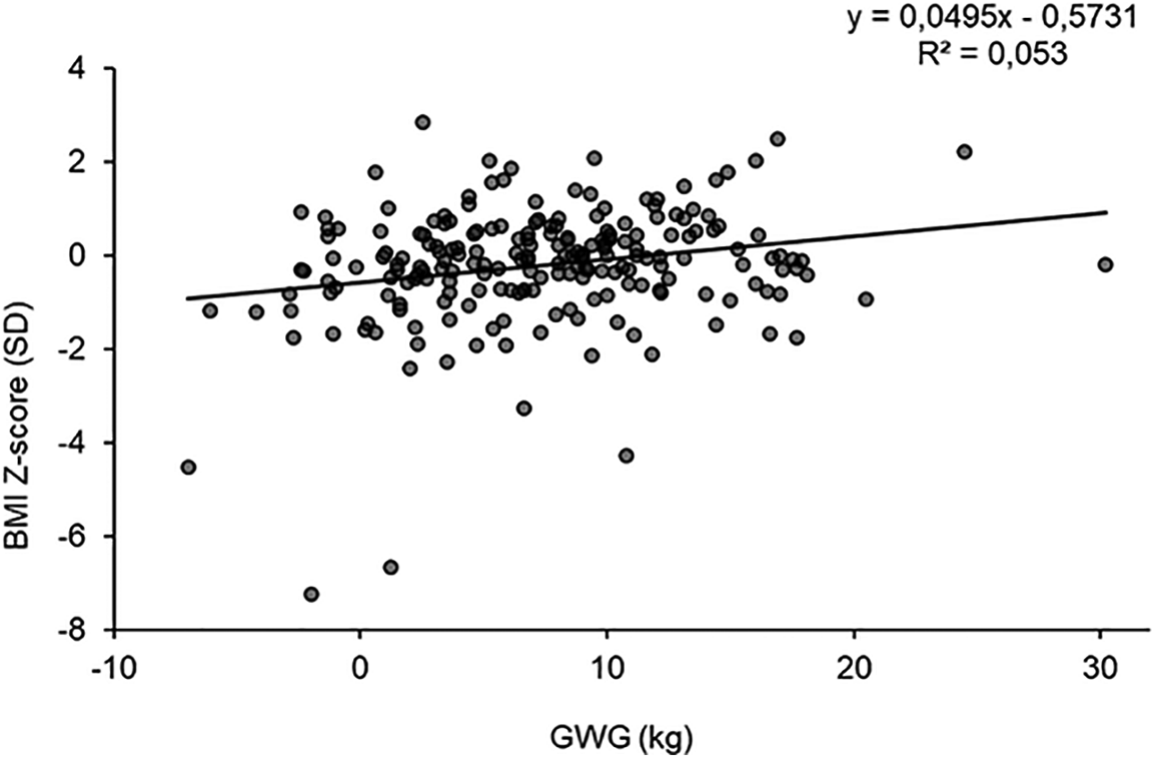
Relationship between GWG and infant BMI-for-age Z-score. The linear regression model is unadjusted.

Gestational weight gain was positively associated with infant birthweight and BMI z-score. Every 1 kg increase in GWG was associated with 16 g greater birthweight (95% CI: 5.34–26.04; *P* = 0.003) and 0.03 SD greater BMI z-score (95% CI: 0.01–0.06; *P* = 0.019) (Table 3). However, the relationship between GWG and BMI z-score was rather weak, with GWG explaining <3% of the total variance in BMI z-score (Figure 1). Additionally, every 1 kg increase in GWG was associated with increased abdominal circumference by 0.06 cm (95% CI: 0.00–0.11; *P* = 0.039) and 0.03 SD greater weight z-score (95% CI: 0.01–0.05; *P* = 0.004).

**Table 3:** Associations between GWG and infant anthropometric outcomes.

	*N*	Estimate (95% CI)	*P*-value
Length (cm)	207	0.04 (0.0;0.08)	0.108
Weight (g)	207	15.69 (5.34;26.04)	0.003
Length z-score	207	0.02 (0.00;0.05)	0.108
Weight z-score	207	0.03 (0.01;0.05)	0.004
BMI z-score	207	0.03 (0.01;0.06)	0.019
Head circumference (cm)	201	0.03 (0.00;0.07)	0.082
Abdominal circumference (cm)	192	0.06 (0.00;0.11)	0.039
Arm circumference (cm)	122	0.06 (0.02;0.10)	0.004
Thigh circumference (cm)	124	0.02 (−0.03;0.08)	0.312

Data are presented as regression coefficients with 95% confidence intervals (95% CI). The analysis is adjusted for maternal pre-pregnancy BMI, parity, and infant gestational age and sex (except for z-scores, which are not adjusted for sex as z-scores are already stratified by sex).

## Discussion

The main finding of our research is that women with pre-pregnancy overweight or obesity who limited their GWG below the IOM recommendation of a maximum of 9 kg gave birth to infants with lower birthweight but similar BMI z-score compared with women exceeding the IOM recommendations. Additionally, a GWG within the IOM recommendation reduced the incidence of emergency cesarean among women with pre-pregnancy overweight or obesity.

The mean GWG among the women who met the IOM-recommended maximum of 9 kg was 3.8 kg. When women were classified into quartiles based on the observed GWG, those in Q1 experienced much lower GWG (range from −7.0 to 3.2 kg; mean 0.2 kg) and gave birth to infants with lower birthweight, BMI z-score, weight z-score, and abdominal circumference compared with women in Q2, Q3, and Q4. However, the incidence of small-for-gestational-age was significantly higher in Q1 compared with Q2 and Q4, but not Q3. We did not find any significant differences in infant outcomes born by mothers who gained from 3.3 kg up to 30.2 kg (i.e., women in Q2, Q3, and Q4) and, in fact, all infants were, on average, within −1 and +1 SD in BMI-for-age and weight z-scores. Thus, in this high-risk group of prospective mothers, focusing on eating a healthy diet during pregnancy may be more important for infant outcomes than focusing on GWG *per se*.

High birthweight has been associated with an increased risk of obesity and diabetes later in life (11). Our study found positive associations of GWG with infant birthweight, BMI z-score, weight z-scores, and abdominal circumference. In particular, for every 1 kg increase in maternal GWG, infant birthweight increased by 16 g. This is in line with the Agency for Healthcare Research and Quality review, which found that for every 1 kg increase in GWG, birthweight increased by 16.7–22.6 g across all BMI categories (23). However, only one study among women with obesity was included in that review, which found that a 1 kg increase in GWG was associated with an 11 g increase in birthweight (24). Similar results were obtained in a cohort study including 146,894 Swedish mothers and their sons (5) finding a positive association between GWG and birthweight, with the association being stronger for women with normal BMI than those with overweight (increased birthweight by 30 g vs. 17 g, respectively, per 1 kg greater GWG) (5). In two cohort studies (6, 25) including infants born by women with normal body weight, greater GWG was also associated with increased infant birthweight. These findings collectively indicate that the strength of the association between GWG and anthropometric outcomes of infants at birth in the present study is in line with what has been observed previously among women with overweight or obesity.

Despite several reviews concluding that excessive GWG is associated with an increase in infant birthweight, recent large and well-designed randomized controlled trials of lifestyle interventions during pregnancy aiming at reducing GWG have all consistently failed (26) or demonstrated only a small reduction in birthweight, despite achieving a significant reduction in GWG (by 1–2 kg) (27). This implies either that the reduction in GWG was not large enough to drive favorable changes in infant anthropometric outcomes, or that such interventions may need to be initiated earlier, i.e., before pregnancy. It is important to note that women with overweight or obesity in these studies still gained considerably more weight during pregnancy than the IOM recommendations (25).

Only 38% of the women participating in our study had a GWG above the IOM recommended maximum of 9 kg, which is considerably lower than the 58% reported in the general population of Danish women with pre-pregnancy obesity (13). We speculate this is likely because women who participated in our study received group-based dietary sessions and individual dietary consultations during pregnancy with a focus on a healthy diet, regardless of the randomization arm.

We found a lower incidence of emergency cesarean in women with GWG below the maximum recommended of 9 kg compared with those with GWG above 9 kg. However, the incidence of small-for-gestational-age was significantly higher in women with GWG less than 3.3 kg. Most previous studies report a lower incidence of cesarean deliveries and pre-eclampsia among women with obesity who gain less weight than the IOM guidelines or, in fact, lose weight during pregnancy; but either no or small effects in the incidence of infants who have low birthweight or are small-for-gestational-age (28–32). Observational studies repeatedly provide evidence of a strong association between lower GWG and increased risk of small-for-gestational-age, especially among women with underweight or normal weight; and also of a strong association between higher GWG and increased risk of large-for-gestational-age, particularly among women with overweight or obesity (12). In our study, we did not observe an association between higher GWG and increased risk of large-for-gestational-age, which is likely because women ate healthy diets and overall restricted their weight gain during pregnancy to ∼7 kg, i.e., within IOM recommendations.

Our results and their interpretation are not without limitations. Our study is a secondary analysis of a randomized controlled trial and thus cannot establish causal relationships between maternal GWG and infant anthropometric outcomes. Moreover, the number of participants may influence the outcome as the study was powered for the APPROACH trial. Maternal pre-pregnancy weight was based on self-reported measurements, which can lead to errors in the estimation of GWG. However, validation studies of pre-pregnancy weight and BMI suggest that self-report is a reasonably accurate source of information (33). In the evaluation of GWG, we did not assess for edema, however, none of the women reported edema as an adverse event during the study(18). Furthermore, we analyzed data from singleton pregnant women with overweight or obesity (BMI 28–45 kg/m^2^), who participated in a diet intervention study during pregnancy. Participants received dietary consultations focused on limiting their GWG and may therefore have been actively interested and invested in better pregnancy outcomes than the average free-living pregnant women with obesity. Furthermore, both diets in the APPROACH study were lower in terms of the glycemic index than the usual diets at baseline for a Danish population (34), which limits the generalizability of the results of our study.

## Conclusion

In conclusion, our results indicate that GWG below the maximum IOM-recommended GWG for women with overweight or obesity was associated with lower birthweight. However, limiting GWG below the IOM recommended range was associated with even lower birthweight, BMI z-score, weight z-score, and infant abdominal circumference. Longitudinal studies of body composition and adiposity indices from infancy to childhood and adulthood are necessary to better understand the importance of GWG in affecting the health of future generations.

## Data Availability

The original contributions presented in the study are included in the article/Supplementary Material, further inquiries can be directed to the corresponding authors.
